# Supplementary material for: Fibrosis Severity in MASLD Determines the Predictive Value of Lp-PLA2 for Carotid Atherosclerosis in Type 2 Diabetes: A Cross-Sectional Study
Source: Biomedicines. 2025 Oct 5;13(10):2431. doi: 10.3390/biomedicines13102431 (PMC12561132; doi:10.3390/biomedicines13102431)

Supplementary Table S1. Logistic regression analysis of Lp-PLA2 quartile levels and metabolic-dysfunction-associated steatotic liver disease in the total population.

	OR (95% CI)				*p for trend*
	Q1	Q2	Q3	Q4	
Model 1	1	1.24 (0.88-1.74)	1.49 (1.05-2.10)	1.61 (1.14-2.28)	0.004
Model 2	1	1.24 (0.87-1.75)	1.39 (0.98-1.99)	1.47 (1.03-2.10)	0.025
Model 3	1	1.27 (0.86-1.87)	1.49 (0.98-2.22)	1.40 (1.03-1.90)	0.043

Q1 to Q4 correspond to the first to fourth quartile groups of Lp-PLA2 levels, with Q1 as the reference group.

Model 1: The unadjusted model.

Model 2: Adjusted for age and sex.

Model 3: Adjusted for age, sex, and BMI.

Supplementary Table S2. Clinical characteristics of all patients grouped by the presence or absence of CAS.

	Without CAS N=331	With CAS N=764	*p*
Age (year)	46.3±12.9	58.5±10.8	<0.001
Male (n, %)	195 (58.9)	488 (63.9)	0.137
Duration (month) †	24 (2-72)	60 (12-132)	<0.001
Body mass index (kg/m^2)	25.3± 3.7	24.6± 3.2	0.002
Waist circumstance (cm)	91.2±10.1	90.4± 9.1	0.296
Hypertension (n, %)	81 (24.5)	347 (45.4)	<0.001
Systolic blood pressure (mmHg)	128±16	134±20	<0.001
Diastolic blood pressure (mmHg)	82±10	82±12	0.492
Smoking (n, %)	77 (23.3)	244 (31.9)	0.005
Use of statins (n, %)	28 (8.5)	131 (17.1)	<0.001
Use of antiplatelet drugs (n, %)	15 (4.5)	117 (15.3)	<0.001
Alanine aminotransferase (U/L) †	26 (17-38)	22 (15-30)	<0.001
Aspartate aminotransferase (U/L) †	21 (18-28)	21 (17-26)	0.228
Uric acid (μmol/L)	368±107	363±101	0.433
eGFR (mL/min/1.73m^2)	106.8±17.7	91.8±20.6	<0.001
Total cholesterol (mmol/L)	5.25±1.39	5.23±1.49	0.811
Triglycerides (mmol/L) †	1.69 (1.24-2.72)	1.49 (1.07-2.16)	<0.001
HDL-c (mmol/L)	1.08±0.26	1.12±0.29	0.014
LDL-c (mmol/L)	3.35±0.86	3.37±1.07	0.708
Fasting blood glucose (mmol/L)	9.4±3.3	8.7±3.3	0.001
HbA1c (%)	10.0±2.1	9.8±2.3	0.149
TyG index	9.52±0.79	9.23±0.75	<0.001
Lp-PLA2 (U/L)	567±158	557±170	0.365
Use of diabetes drugs			
Metformin (n, %)	102 (30.8)	314 (41.1)	0.002
Insulin secretagogues (n, %)	47 (14.2)	188 (24.6)	<0.001
Insulin (n, %)	29 (8.8)	146 (19.1)	<0.001
Metabolic syndrome (n, %)	219 (72.3)	508 (73.7)	0.690
MASLD (n, %)	230 (69.5)	444 (58.1)	<0.001
DKD (n, %)	36 (10.9)	200 (26.2)	<0.001
ASCVD (n, %)	10 (3.0)	112 (14.7)	<0.001

†Continuous variables are expressed as the median with the IQR for a non-Gaussian distribution. Abbreviation: CAS, carotid atherosclerosis; eGFR, estimated glomerular filtration rate; HDL-c, high-density lipoprotein-cholesterol; LDL-c, low-density lipoprotein-cholesterol; TyG index, triglyceride–glucose index; Lp-PLA2, lipoprotein-associated phospholipase A2; MASLD, metabolic-dysfunction-associated steatotic liver disease; DKD, diabetic kidney disease; ASCVD, atherosclerotic cardiovascular disease.

Supplementary Table S3. Clinical characteristics of all patients grouped by the presence or absence of CAS (after matching).

	Without CAS N=287	With CAS N=287	*P*
Age (year)	51.8±10.8	52.6±11.6	0.42
Male (n, %)	161 (56.1)	143 (49.8)	0.21
Duration (month) †	24（5-96）	24（6-90）	0.96
Body mass index (kg/m^2)	24.8±3.2	24.9±3.6	0.777
Waist circumstance (cm)	90.5±9.2	89.8±9.6	0.428
Hypertension (n, %)	87 (30.5)	115 (39.9)	0.047
Systolic blood pressure (mmHg)	129±16	133±20	0.023
Diastolic blood pressure (mmHg)	82±11	84±12	0.082
Smoking (n, %)	59 (20.6)	69 (24.2)	0.427
Use of statins (n, %)	35 (12.1)	32 (11.2)	0.883
Use of antiplatelet drugs (n, %)	16 (5.8)	22 (7.6)	0.571
Alanine aminotransferase (U/L) †	24(17-33)	22(16-32)	0.204
Aspartate aminotransferase (U/L) †	21(18-26)	21(17-26)	0.996
Uric acid (μmol/L)	350±100	354±108	0.707
eGFR (mL/min/1.73m^2)	102.0±17.2	97.2±20.2	0.007
Total cholesterol (mmol/L)	5.20±1.49	5.43±1.49	0.104
Triglycerides (mmol/L) †	1.60 (1.24-2.49)	1.54 (1.19-2.21)	0.212
HDL-c (mmol/L)	1.10±0.27	1.12±0.26	0.535
LDL-c (mmol/L)	3.31±0.90	3.52±1.08	0.022
Fasting blood glucose (mmol/L)	9.4±3.5	9.2±3.6	0.468
HbA1c (%)	9.9±2.1	10.1±2.3	0.491
TyG index	9.46±0.79	9.33±0.77	0.073
Lp-PLA2 (U/L)	554±156	563±168	0.534
Use of diabetes drugs			
Metformin (n, %)	104 (36.3)	96 (33.6)	0.620
Insulin secretagogues (n, %)	46 (16.1)	59 (20.6)	0.271
Insulin (n, %)	32 (11.2)	44 (15.2)	0.264
Metabolic syndrome (n, %)	198 (69.0）	213 (74.1）	0.303
MASLD (n, %)	183 (63.7)	184 (64.1)	0.998
DKD (n, %)	32 (11.2)	61 (21.1)	0.007
ASCVD (n, %)	10 (3.6)	22 (7.6)	0.100

†Continuous variables are expressed as the median with the IQR for a non-Gaussian distribution. Abbreviation: CAS, carotid atherosclerosis; eGFR, estimated glomerular filtration rate; HDL-c, high-density lipoprotein-cholesterol; LDL-c, low-density lipoprotein-cholesterol; TyG index, triglyceride–glucose index; Lp-PLA2, lipoprotein-associated phospholipase A2; MASLD, metabolic-dysfunction-associated steatotic liver disease; DKD, diabetic kidney disease; ASCVD, atherosclerotic cardiovascular disease.

Supplementary Table S4. The relationship between Lp-PLA2 levels at the cutoff value intervals and CAS.

	OR (95%CI)	*P*
< 570U/L (per SD)	1.06 (0.69-1.62)	0.81
> 570U/L (per SD)	2.67 (1.31-5.42)	0.007

Model adjusted for age, sex, disease duration, systolic blood pressure, smoking, glycated hemoglobin, statin use, and diabetic nephropathy.

Supplementary Figure S1. Comparison of Lp-PLA2 among different metabolic-dysfunction-associated steatotic liver disease (MASLD) subgroups based on different degrees of steatosis measured via CT (A) and different degrees of fibrosis measured via FIB-4 score (B) and the corresponding scatter plot between Lp-PLA2 and FIB-4 (C)

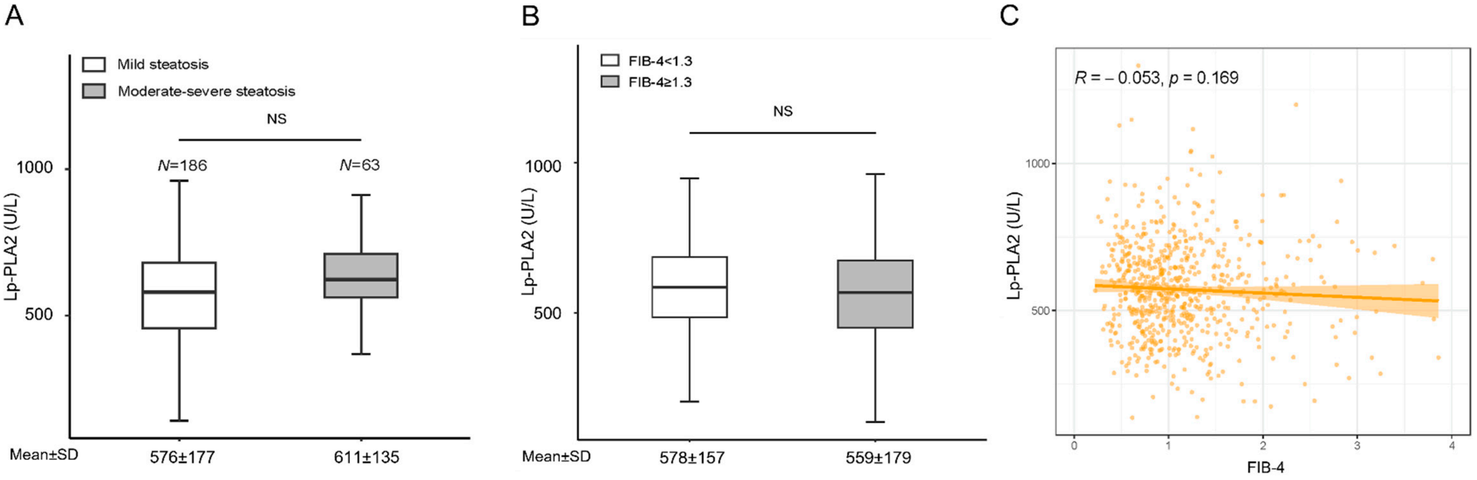

Supplement: Supplementary file 1 [file biomedicines-13-02431-s001.zip › biomedicines-3811707-supplementary.pdf]
